# Perceived Complexity as Normalized, Integrated, Localized Shannon Entropy

**DOI:** 10.3390/e28030279

**Published:** 2026-03-01

**Authors:** Sébastien Berquet, Norberto M. Grzywacz

**Affiliations:** 1 Department of Biology, Loyola University Chicago, Chicago, IL 60660, USA; sberquet@luc.edu; 2 Department of Biomedical Engineering, Johns Hopkins University, Baltimore, MD 21218, USA; 3 Department of Psychology, Loyola University Chicago, Chicago, IL 60660, USA

**Keywords:** perceived complexity, Shannon entropy, image statistics, natural images, urban images, artistic paintings, aesthetics, color, luminance, spatial scales

## Abstract

Perceived complexity is a key component of sensory brain function as it indicates the number of resources necessary to process incoming information. A recently proposed measure of perceived complexity defined it as normalized Shannon entropy. However, the proposal used probability distributions estimated from the entire sensory signal at once. Here, we first used synthetically created images and abstract expressionism art to show that using such distributions seemed incompatible with perceived complexity. This incompatibility persisted even if we performed the calculations at different scales, that is, in multiple image resolutions. We then proposed an alternate theory that postulated that perceived complexity arose from the integration of localized Shannon entropy. The outcome of this integration was then normalized to define an index of complexity. We measured this index and integrated Shannon entropy in 704 images obtained from natural and urban settings, painted by well-known artists, or created synthetically. Moreover, we studied the dependence of these measurements on the spatial scale used to measure Localized Shannon Entropy. We found that normalized, integrated, localized Shannon entropy at low spatial scales is consistent with the phenomenology of perceived complexity and illustrates interesting aesthetic choices of different artists.

## 1. Introduction

People get pleasure when seeing the details of a piece of art or listening to the variations in music. These details convey complexity, which as a source of information [1,2,3] is a major aesthetic value in human endeavors [4,5,6,7,8]. The significance of complexity is in the delivery of the amount of details to the brain, that is, how much information is present in the stimulus [1]. The brain uses this to allocate resources for handling the incoming sensory signal [1,6]. Hence, investigators have labored to comprehend how the brain computes complexity. In doing so, they have built definitions of complexity, including, for example, the number of “objects” a stimulus has and a perceptual scale [4,9,10,11]. Another definition has used information theory [1,12,13]. To be exact, this definition uses a normalized version of Shannon entropy [1,6,14]. But different versions of complexity as normalized Shannon entropy are possible [15], and in this article, we examine whether the main ones are compatible with perceived complexity.

Here, we will focus on complexity measures for visual details for the sake of simplicity. Analyses of complexity using information theory are similar for other sensory modalities, such as for example, in music [3,16,17]. But we will focus on the complexity of static images to reduce the number of variables that we must quantify [18,19,20]. We will work with two main variables, namely, intensity and color [6,18]. We will focus on them in isolation, that is, pixel by pixel and in pairs of pixels to analyze spatial complexity [14,21,22].

We classify the main ways to build normalized Shannon entropy measures of visual complexity into globalized and localized. By globalized, we mean measures that use the distributions of a measurement, for example, intensity, across the entire image. We then use these distributions to obtain the Shannon entropies. In turn, by localized, we mean the process of measuring the entropies in small subregions of the image and then integrating these partial entropies [23,24,25]. Because measuring entropy involves a nonlinearity, the globalized and localized methods yield different results. We thus hypothesize that if one uses globalized entropy to measure complexities, then they would be different for some classes of images than those obtained with localized entropy.

We think of the small subregions in the computation of Localized Shannon Entropy as spatial scales. The human visual system processes information across multiple spatial scales, from fine details (high spatial frequencies) to broad layouts (low spatial frequencies) [26,27,28,29]. This processing uses specialized neural channels that efficiently integrate coarse and fine information [30,31,32,33]. Visual processing shifts from early visual areas for details to higher-level areas for large-scale space [34,35,36]. In this article, we not only use spatial scales for Localized Shannon Entropy but also for its globalized version. The difference is that in Globalized Shannon Entropy, we first make the image coarser according to the scale and then compute the overall entropy.

To test how well Localized and Globalized Shannon Entropies align with perceived complexity, we must use appropriate images. We begin with synthetically created images controlling complexity and other visual variables precisely [37,38]. We also use urban and natural images employed in a recent study of Fisher information [6]. To complement these images, we use artistic paintings whose range of complexities could help us probe the various proposed measures of entropy. We begin with Giorgione, who as a High Renaissance artist from the Venetian School has “realistic” figurative paintings. Consequently, they may be closest to our natural and urban images in statistical terms [39,40]. We also include abstract expressionist paintings from Jackson Pollock’s drip period (1947–1952) because they appear complex to the eye [41,42]. At the other end of the spectrum, we have Pablo Picasso in his Period of Cubism (1909–1919—[43,44]), Matisse in his period of bold simplification (1930–1941—[45,46]), and late Joan Miró paintings (1960–1983—[47,48]). These artists during these periods have paintings with large areas without details and thus, with lower spatial complexity. Finally, we use paintings produced by Claude Monet for the four Impressionist Exhibitions (1872–1882—[49,50,51]). Although these paintings are figurative, they have blurred borders, possibly reducing spatial complexity in a way that is different from Picasso and Matisse.

In the rest of this article, we first develop mathematically all the complexity measures that we are testing here (Section 2). Next, we describe the images in detail and the statistical tests used to compare the complexity measures (Section 3). The results (Section 4) progress from Globalized (Section 4.1) to Localized Complexities (Section 4.2, Section 4.3 and Section 4.4). We finish with the discussion (Section 5). In it, we address localized and globalized computations, the problem of spatial scales, the structure of images, the distinctions between different types of images, and a comparison of art and nature.

## 2. Theory

We divide this section into two parts: The first presents a general description of the theoretical considerations, while the second introduces their mathematical formulations. Therefore, Section 2.1 has a verbal explanation of the concepts and theoretical results without equations. Our aim with that subsection is to help the reader understand the theoretical issues at a more intuitive level. That subsection may allow readers initially to avoid the Section Mathematical Formulations (Section 2.2). These readers may be able jump straight to the results (Section 4).

### 2.1. Overview of the Theoretical Ideas

A recently proposed measure of perceived complexity defined it as normalized Shannon entropy [6,15]. Hence, that proposal interpreted entropy in the information-theoretic sense. Thus, entropy was a measure of the unpredictability or variability of a probability distribution. When applied to image properties such as intensity or hue, this entropy quantified the uncertainty associated with these measurements across the image. Here, higher entropy values corresponded to greater variability in pixel values and thus to a larger amount of information required to encode or process the visual signal. For example, a random-noise image would yield high entropy, whereas a uniform image would yield zero entropy.

The aforementioned proposal to measure perceived complexity as normalized Shannon entropy used probability distributions estimated from the entire sensory signal at once. Thus, the computations would first sample the distribution of intensities in all the pixels of an image. Then, the computation would get the Shannon entropy from this distribution. Normalization would then proceed by dividing the result of the computation by the maximally possible Shannon Entropy. This maximal entropy would be computed from sensory signals in which all parts would be randomly selected from the distribution of all possible values (intensities). This normalization by maximal entropy makes complexity an index from 0 to 1. Here, we refer to this measure of complexity as the Normalized, Globalized Shannon Entropy or Globalized Complexity.

We also studied Globalized Complexity for different pixel sizes. Thus, we subdivided the image into non-overlapping square regions with equal areas. For each of these squares, we got the median of the intensities and the circular median of the hues. These medians gave rise to new distributions, which we used to calculate Normalized, Globalized Shannon Entropy. We then plotted these entropies as a function of the sides of the squares, which are called spatial scales.

Next, we switched our attention to Normalized, Integrated, Localized Shannon Entropy or Localized Complexity. The difference between this type of complexity and the globalized one was that for the local version, we compute entropy in the non-overlapping squares. (For Globalized Complexity, we computed their medians.) We then added all the resulting entropies and normalized the outcome. And as for the globalized case, we also plotted Localized Complexities as a function of scales. In these plots, we use as a parameter for the curves the number of bins used to estimate the probability distributions because it could affect the entropy and thus, the complexity.

In this article, we focus on visual complexity for pixelized images for the sake of simplicity and without loss of generality. The Normalized Shannon Entropy definitions of visual complexity begin with the distributions of measurable variables from images. For example, luminance complexity uses the Shannon Entropy of the distribution of image luminance [6,14,22]. In turn, chromatic complexity uses the distribution of hue [6]. A more complicated type of complexity is the spatial one [1,14,21]. It uses the two-dimensional distribution of the measurable values, that is, the probability that a point has a value while another point has a different value.

### 2.2. Mathematical Formulations

Let m be a measurement in a pixel of the image (for example, intensity or hue). Let the set of possible values that m can attain be $\left\{m_{1},\cdots ,m_{k},\cdots m_{M}\right\}$ (for example, in an 8-bit RGB image, M=256 for the red gun). Consider an N×N Image I. The possible measurements m in its various pixels can be written as the matrix

(1) $$m(I)=\left(\begin{array}{ccc} m_{11}\left(I\right) & \cdots & m_{1N}\left(I\right)\\ \vdots & m_{ij}\left(I\right) & \vdots \\ m_{N1}\left(I\right) & \cdots & m_{NN}\left(I\right) \end{array}\right)$$

Let the kth possible value of the measurement m among the possible M occur $M_{k}\left(I\right)$ times among the $N^{2}$ measurements in m(I). Then, the sampled probability of this kth value given Image I is

(2) $$P_{m}^{\left(1\right)}\left(k|I\right)=\frac{M_{k}\left(I\right)}{\sum_{j=1}^{M}M_{j}\left(I\right)}=\frac{M_{k}\left(I\right)}{N^{2}},\;$$

where the Symbol “(1)” marks Complexity of Order 1, that is, taking the measurements from one point at a time. From Equation (2), we define the sampled Globalized Shannon Entropy of Order 1 of Measurement m for Image I as

(3) $$H_{m}^{\left(1\right)}\left(I\right)=-\overset{M}{\underset{k=1}{\sum }}P_{m}^{\left(1\right)}\left(k|I\right)\log_{2}\left(P_{m}^{\left(1\right)}\left(k|I\right)\right).\;$$

For the sake of simplicity, we also call this quantity the Globalized Entropy. In practice, if $P_{m}^{\left(1\right)}\left(k|I\right)=0$ for a k, the term is not included in the sum, avoiding the singularity of the logarithm. This is possible because $\underset{y\to 0}{\lim}\;y\;\log\;y=0$.

To create an index of complexity out of this entropy, we divide it by its largest possible value given any arbitrary image. This largest entropy comes from images for which every point has a measurable value randomly picked from all possible values. Thus, $P_{m}^{\left(1\right)}\left(k|I\right)=1/M$. Substituting this result for $P_{m}^{\left(1\right)}$ in Equation (3), one gets the maximal Entropy of Order 1:

(4) $$H_{max,1}\left(m\right)=\log_{2}\left(M\right)\;.$$

Dividing Equation (3) by Equation (4), gives the Globalized Complexity of Order 1 as the Normalized Shannon Entropy:

(5) $$C_{m}^{\left(1\right)}\left(I\right)=\frac{H_{m}^{\left(1\right)}\left(I\right)}{\log_{2}\left(M\right)}\;.\;$$

Again, for the sake of simplicity, we also call this quantity the Globalized Complexity. Because the denominator is $H_{max,1}$, we have $0\leq C_{m}^{\left(1\right)}\left(I\right)\leq 1$. We get 0 for single-tone images (that is, the simplest ones) and 1 for images whose measurable variables spread homogeneously through all possible values.

Complexity of Order 2 (Spatial Complexity) is like Complexity of Order 1, except that we now compare pairs of image points. We begin by defining the Entropy of Order 2 for the Measurement m, Isometric Transformation T, and Image I. For this definition, we first get $M_{l_{1}l_{2}}^{\left(T\right)}\left(I\right)$. This measurement is the number of times an image point with the $l_{1}$th measurement value is in juxtaposition with a point with the $l_{2}$th value after the transformation. From this number, we define the following conditional probability:

(6) $$P_{m,T}^{\left(2\right)}\left(k_{1},k_{2}\left|I\right.\right)=\frac{M_{k_{1}k_{2}}^{\left(T\right)}\left(I\right)}{\sum_{j_{1}=1}^{M}\sum_{j_{2}=1}^{M}M_{j_{1}j_{2}}^{\left(T\right)}\left(I\right)}\;.$$

From this equation, we define the sampled Globalized Shannon Entropy of Order 2 of Measurement m given Transformation T and Image Q as

(7) $$H_{m,T}^{\left(2\right)}\left(I\right)=-\overset{M}{\underset{k_{1}=1}{\sum }}\overset{M}{\underset{k_{2}=1}{\sum }}P_{m,T}^{\left(2\right)}\left(k_{1},k_{2}\left|I\right.\right)\log_{2}\left(P_{m,T}^{\left(2\right)}\left(k_{1},k_{2}\left|I\right.\right)\right)\;.$$

The maximal value of Entropy of Order 2 occurs when all pairs $\left(k_{1},k_{2}\right)$ are equally likely. Thus, $P_{m,T}^{\left(2\right)}\left(k_{1},k_{2}\left|I\right.\right)=1/M^{2}$. Substituting this result for $P_{m,T}^{\left(2\right)}$ in Equation (7), one gets the maximal Entropy of Order 2:

(8) $$H_{max,2}\left(m,T\right)=2\log_{2}(M)\;.$$

Dividing Equation (7) by Equation (8), one gets the Globalized Complexity of Order 2 as the Normalized Shannon Entropy:

(9) $$C_{m,T}^{\left(2\right)}\left(I\right)=\frac{H_{m,T}^{\left(2\right)}\left(I\right)}{2\log_{2}\left(M\right)}\;.$$

The Equations (1)–(9) defining entropies and complexities are modified when considering different spatial scales. We define the Spatial Scale s as an integer divisor of N. This scale is used to divide the image into N/s×N/s subregions, transforming the matrix in Equation (1) into

(10) $$m_{\mu }\left(I,s\right)=\left(\begin{array}{ccc} m_{\mu ,1,1}\left(I,s\right) & \cdots & m_{\mu ,1,\frac{N}{s}}\left(I,s\right)\\ \vdots & m_{\mu ,i,j}\left(I,s\right) & \vdots \\ m_{\mu ,\frac{N}{s},1}\left(I,s\right) & \cdots & m_{\mu ,\frac{N}{s},\frac{N}{s}}\left(I,s\right) \end{array}\right)\;,\;\;\;\;\;\;\;\;\;\;\;\;\;\;\;\;\;\;\;\;\;\;\;\;\;\;\;\;\;\;\;$$

where

(11) $$m_{\mu ,i,j}\left(I,s\right)=\mu \left(\begin{array}{ccc} m_{\left(i-1\right)\frac{N}{s}+1,\left(j-1\right)\frac{N}{s}+1}\left(I\right) & \cdots & m_{\left(i-1\right)\frac{N}{s}+1,j\frac{N}{s}}\left(I\right)\\ \vdots & m_{\left(i-1\right)\frac{N}{s}+l,\left(j-1\right)\frac{N}{s}+n}\left(I\right) & \vdots \\ m_{i\frac{N}{s},\left(j-1\right)\frac{N}{s}+1}\left(I\right) & \cdots & m_{i\frac{N}{s},j\frac{N}{s}}\left(I\right) \end{array}\right),\;$$

with μ being a measurement, that is, a statistic of the submatrix of $m\left(I\right)$ (Equation (1)) indicated in Equation (11). The statistic μ can be the mean, median, variance, entropy, or any other relevant measurement. In this article, we use μ=median when calculating Globalized Complexities as a function of scale. Hence, we substitute Equation (10) for Equation (1) in the calculation of Equations (2)–(9) to get $C_{m}^{\left(1\right)}\left(s,\mu =\mathrm{med},I\right)$ and $C_{m,T}^{\left(2\right)}\left(s,\mu =\mathrm{med},I\right)$.

The alternative considered in this article is Localized Complexity. This alternative begins with Equations (10) and (11) but this time, μ=entropy. Consequently, we develop equations like Equations (2) and (3) but for matrices as in Equation (11). With these new equations, Equation (10) becomes the following for Orders 1 and 2, respectively,

(12) $$m_{\mu }^{\left(1\right)}\left(I,s\right)=\left(\begin{array}{ccc} H_{m,1,1}^{\left(1\right)}\left(I,s\right) & \cdots & H_{m,1,\frac{N}{s}}^{\left(1\right)}\left(I,s\right)\\ \vdots & H_{m,i,j}^{\left(1\right)}\left(I,s\right) & \vdots \\ H_{m,\frac{N}{s},1}^{\left(1\right)}\left(I,s\right) & \cdots & H_{m,\frac{N}{s},\frac{N}{s}}^{\left(1\right)}\left(I,s\right) \end{array}\right)$$

and

(13) $$m_{\mu ,T}^{\left(2\right)}\left(I,s\right)=\left(\begin{array}{ccc} H_{m,T,1,1}^{\left(2\right)}\left(I,s\right) & \cdots & H_{m,T,1,\frac{N}{s}}^{\left(2\right)}\left(I,s\right)\\ \vdots & H_{m,T,i,j}^{\left(2\right)}\left(I,s\right) & \vdots \\ H_{m,T,\frac{N}{s},1}^{\left(2\right)}\left(I,s\right) & \cdots & H_{m,T,\frac{N}{s},\frac{N}{s}}^{\left(2\right)}\left(I,s\right) \end{array}\right),\;$$

where $H_{m,i,j}^{\left(1\right)}\left(I,s\right)$ and $H_{m,T,i,j}^{\left(2\right)}\left(I,s\right)$ are the entropies of the matrix in Equation (11). To get the total Localized Entropies of Orders 1 and 2, we sum the partial entropies in Equations (12) and (13) [23,24,25] to get, respectively,

(14) $$H_{m}^{\left(1\right)}\left(I,s\right)=\overset{\frac{N}{s}}{\underset{i=1}{\sum }}\overset{\frac{N}{s}}{\underset{j=1}{\sum }}H_{m,i,j}^{\left(1\right)}\left(I,s\right)$$

and

(15) $$H_{m,T}^{\left(2\right)}\left(I,s\right)\left(I,s\right)=\overset{\frac{N}{s}}{\underset{i=1}{\sum }}\overset{\frac{N}{s}}{\underset{j=1}{\sum }}H_{m,T,i,j}^{\left(2\right)}\left(I,s\right).$$

Finally, we must divide the entropies in Equations (14) and (15) by their maximal values to get the respective complexities. These maxima are equal to the maximum entropies in each region times the number of regions, giving, respectively,

(16) $$H_{max,1}\left(m,I,s\right)={\left(\frac{N}{s}\right)}^{2}\log_{2}\left(M\right)$$

and

(17) $$H_{max,2}\left(m,I,s\right)=2{\left(\frac{N}{s}\right)}^{2}\log_{2}\left(M\right).$$

Therefore, dividing Equations (14) and (16) by Equations (16) and (17), respectively, gives the following Localized Complexities of Orders 1 and 2:

(18) $$C_{m}^{\left(1\right)}\left(I,s\right)=\frac{s^{2}H_{m}^{\left(1\right)}\left(I,s\right)}{N^{2}\log_{2}\left(M\right)}$$

and

(19) $$C_{m,T}^{\left(2\right)}\left(I,s\right)=\frac{s^{2}H_{m,T}^{\left(2\right)}\left(I,s\right)\;}{2N^{2}\log_{2}\left(M\right)}.\;$$

An important consequence of these equations is that if pixels were independent in an image, localized entropies would fall with the square of the spatial scale (Equations (16) and (17)), while localized complexities would stay constant (Equations (18) and (19)). Any deviations of these relationships are due to image structures.

## 3. Materials and Methods

### 3.1. Images

In this study, we used 3232 images, of which we analyzed 704 fully. The synthetic images were created with a resolution of 300 × 300 pixels according to the algorithm described by Mather, Rhee, and their colleagues [37,38]. The other images had their size first reduced with a bicubic-interpolation method [52] so that the smallest dimension became 300 pixels. We then cropped the other dimension in the middle 300 pixels, so the images in this study were all square and with the same size. The lateral size of 300 was chosen to remain consistent with the synthetic images of Mather, Rhee, and colleagues. This size also ensured methodological consistency across all images. Standardizing images to a fixed dimension avoided confounds related to varying image size, aspect ratio, or sampling density, all of which could directly affect entropy estimation. Additionally, 300 had eighteen integer divisors, allowing for a systematic and comprehensive study of spatial scales within a single framework.

The 1000 natural and urban images that we used in our study were from a previous publication [6]. We refer the reader to it for the technical details of how we obtained these images. In turn, the 1800 synthetic images were like those of Mather, Rhee, and colleagues. The images were designed to control the complexity and symmetry of the images. The complexity was controlled by setting the radius of local integration. In turn, the symmetry was the probability that pixels with the same ordinates and equidistant from the central axis had the same values. However, different from Mather, Rhee, and colleagues, our pixels could only have the following RGB intensities: (248 0 0), (62 0 0), (0 248 0), and (0 62 0). Therefore, the pixels of our synthetic images could only be red or green, with either high or low intensities. Another difference with the images of Mather, Rhee, and colleagues is that we only used degrees of symmetry of 0, 0.5, and 1, and radii of integrations of 1, 2, 4, 8, 16 and 32. Finally, we used 432 artistic paintings that were available as part of the public domain on the internet. The artistic paintings came from Artchive (https://www.artchive.com/, accessed on 24 February 2026). The artists and periods used were Giorgione (1500–1510), Claude Monet (1872–1882), Matisse (1930–1941), Pablo Picasso (1909–1919), Joan Miró (1960–1983), and Jackson Pollock (1947–1952). (The introduction has a justification for these artists and periods.) We constrained the periods to avoid unintentional variations in the statistics of the images. All the images are available at the Supplementary Information at https://osf.io/nwz42 (accessed on 24 February 2026), and we provide a user-friendly way to view them at https://sberquet.github.io/Perceived-Complexity-As-Normalized-Integrated-Local-Shannon-Entropy/(accessed on 24 February 2026).

### 3.2. Quantitative Analysis

We used a MATLAB (MATLAB Version R2025b, Mathworks, Natick, MA, USA) code written in-house to obtain and statistically analyze the eight complexity and entropy types for each image (Equations (3), (5), (7), (9), (14), (15), (18) and (19). To calculate spatial complexities and entropies, we used the 1-pixel-translation transform, as done and argued for in previous studies [6,15,22]. To increase statistical quality, we used both horizontal and vertical translations in the calculation of spatial complexity and entropy. Furthermore, we plotted complexities and entropies as a function of spatial scale. The scales used for Globalized Complexity were 1, 2, 3, 4, 5, 6, 10, 12, 15, 20, 25, 30, 50, and 60 because larger ones did not generate enough subregions to make measurements for an accurate estimation of entropy and thus, complexity. In turn, the range of scales used for the measurement of Localized Complexity was different. Small scales led to subregions that had too few pixels for accurate estimations of entropy. If we considered a scale s, the subregion could lead to $s^{2}$ measurements of a first-order variable (for example, intensity). The same subregion would lead to 2$s\left(s-1\right)$ measurements of a second-order variable with our double 1-pixel-translation transform (spatial intensity in the example). To get good accuracy, we wanted to have the number of these measurements to be at least five times the number of bins in the estimated probability distributions. Therefore, we let this number of bins be ν for a first-order measurement. Then this number was $\nu^{2}$ for its spatial version. Thus, our accuracy requirement meant $5\nu \leq s^{2}$ for the first-order variable and

(20) $$5\nu^{2}\leq 2s\left(s-1\right).$$

for second-order one. This inequality indicated that the spatial scale could not be too small if we wanted a reasonable number of bins. Hence, the scales used for Localized Entropy were 10, 12, 15, 20, 25, 30, 50, 60, 75, 100, 150, and 300. For each of these scales, we used the number of bins allowed by Inequality 20. Thus, the number of bins was determined by a data-adequacy constraint, rather than by standard histogram rules [53,54,55,56,57]. This constraint ensured statistically reliable probability estimation within each subregion. Binning therefore serves as an implementation requirement rather than a conceptual degree of freedom of the localized complexity formulation. At any given spatial scale, all images were evaluated using the same binning configuration, ensuring that comparisons across image classes were performed under identical statistical conditions. 

To test statistically whether two entropies and thus, the corresponding complexities were different, we used bootstrapping [58] accompanied by a Mann–Whitney test [59]. Furthermore, we applied the Benjamini–Hochberg procedure for controlling the False Discovery Rate in multiple hypothesis testing [60]. If instead we had entropies from three or more sets of images or from different types of entropies, we first tested the homogeneity of their medians with a Kruskal–Wallis test [61]. If we detected inhomogeneity, we followed up with a Mann–Whitney post-hoc test. We also used Kruskal–Wallis tests for the null hypothesis that Globalized Complexities did not depend on spatial scale. As before, if we detected dependence, we followed up with Mann–Whitney tests to measure the direction of the trends. Finally, we performed statistical tests on the log–log dependences of entropies and complexities on spatial scales. In these tests, we first performed linear regressions, getting their coefficients of determination. We then tested whether these coefficients and the regression intercepts were different from zero.

The MATLAB code for the quantitative and statistical analyses is in the Supplementary Information at https://osf.io/nwz42 (accessed on 24 February 2026).

## 4. Results

### 4.1. Globalized Complexity

**Figure 1:**
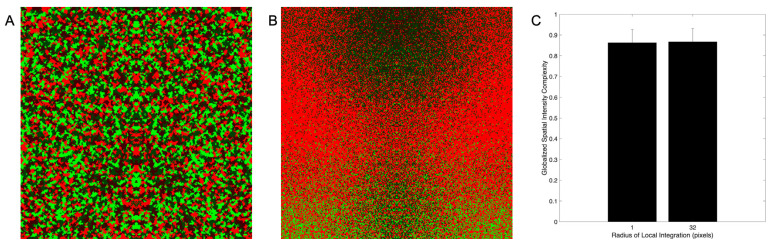
Comparison of globalized spatial-intensity complexity of synthetic images. (**A**). Image with radius of local integration = 1. (**B**). Image with radius of local integration = 32. (**C**). Globalized spatial-intensity complexity for the images in (**A**,**B**). The degree of symmetry in both images is 1 and the error bars are standard deviations. Although the image in Panel (**A**) seems more complex than that in Panel (**B**), their globalized spatial-intensity complexities are statistically indistinguishable.

Our first goal was to test quantitatively whether Normalized, Globalized Shannon Entropy aligns with perceived complexity across different types of images. To conduct this test, we applied Equations (5) and (9) to hundreds of images as described in Section 3.1. The stimuli included natural and urban images, synthetically generated ones, and artistic paintings. The data quickly showed that although Globalized Complexity may work well for natural and urban images, it does not for the synthetic ones. Figure 1 illustrates an example of this failure for Globalized Spatial-Intensity Complexity.

A synthetic image with a radius of local integration of 1 (Figure 1A) appears more complex than another with a radius of 32 (Figure 1B). The former image has small green, red, bright, and dark blobs, while the latter has large red, green, bright, and dark regions. However, the globalized spatial-intensity complexities of these two images are statistically indistinguishable (Figure 1C—bootstrapping and Mann–Whitney test). Why did the globalized entropy not reflect the apparent difference in complexity? We hypothesized that this failure was because the large regions in Figure 1B were not pure but had interspersed pixels of other colors and intensities. This hypothesis predicted that the failure of Globalized Complexity might disappear at larger scales. This is because these scales might integrate out the “noisy” pixels.

**Figure 2:**
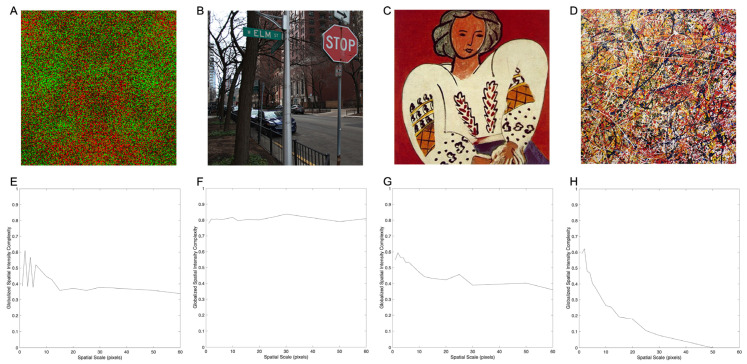
Globalized complexity as a function of spatial scale for different types of images. (**A**). Synthetic image with the radius of local integration = 1 and degree of symmetry = 0.5. (**B**). Urban image. (**C**). Henry Matisse’s La Blouse Roumaine (1940). (**D**). Jackson Pollock’s Drip Painting (1951). (**E**–**H**). Globalized spatial-intensity complexity as a function of spatial scale for the images in panels (**A**–**D**), Respectively. Globalized complexities for Pollock’s painting are lower than those for the urban image across scales.

Consequently, we next tested whether Normalized, Globalized Shannon Entropy at different scales could fix the problem observed in Figure 1. This Globalized Complexity as a function of scale was defined mathematically as $C_{m}^{\left(1\right)}\left(s,\mu =\mathrm{med},I\right)$ and $C_{m,T}^{\left(2\right)}\left(s,\mu =\mathrm{med},I\right)$ after Equation (11). For this purpose, we fixed the images and varied scales from 1 to 60, applying these definitions. We used all the images in our study but decided to show the results of the four that best illustrate the conclusions that we reached. Thus, Figure 2 shows the results for synthetic and urban images and for paintings by Henry Matisse and Jackson Pollock. This figure again plots the Globalized Spatial-Intensity Complexity.

Figure 2A,E support the hypothesis that the Globalized Spatial Complexity at low spatial scales is high because of the inner “noisy” pixels. As seen in Figure 2E, as the scale increased, the “noisy” pixels were integrated out, lowering the complexity. However, this scale dependence did not solve all the problems with Globalized Complexity. Comparing the Jackson Pollock painting with the urban scene showed that the former appeared more complex (Figure 2B,D), but the Globalized Complexity was larger for the latter regardless of scale. Thus, Globalized Complexity did not appear consistent with perceived complexity.

Figure 2 shows four other important properties of Globalized Complexity: First, the urban scene had a particularly high complexity, which was roughly independent of scale (compare Figure 2B with Figure 2A,C,D). (However, we did detect a small statistically significant scale dependence when using bootstrapping and the Kruskal–Wallis test.) Second, the non-urban images produced Globalized Complexity that fell with spatial scale (Figure 2A,C,D). Third, Globalized Complexities fell to zero for Pollock’s painting because, at large scales, a single color or intensity dominated. Fourth, Globalized Complexities were not as small as predicted for Matisse’s painting.

In sum, Figure 1 and Figure 2 suggest that Globalized Complexity is not consistent with how humans perceive complexity.

### 4.2. Localized Complexity

**Figure 3:**
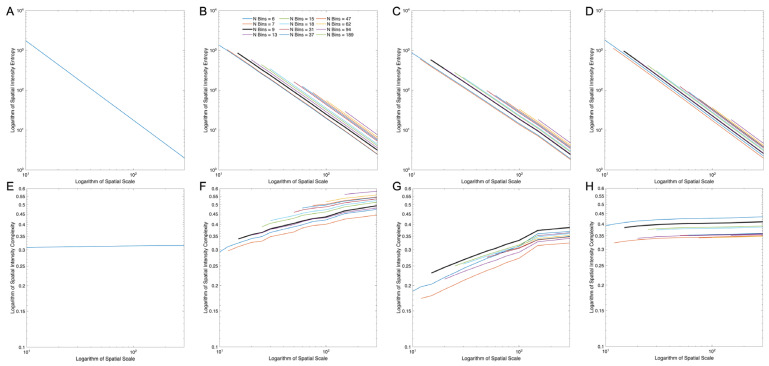
Log–log plots of localized spatial-intensity entropies and complexities as a function of spatial scale for the images in Figure 2. (**A**–**D**). Entropies corresponding to Figure 2A–D, respectively. (**E**–**H**). Complexities corresponding to Figure 2A–D, respectively. In Panels (**B**–**D**,**F**–**H**), the lines correspond to the numbers of bins (NBins) used to get the probabilities of intensity (Panel (**B**) legend; the longer curves represent fewer bins). We bolded the NBins = 9 line since it has the number of bins used later in this article. While the entropies fall as power laws, complexities are either flat or rise slowly.

Given the limitations observed for Normalized, Globalized Shannon Entropy, we next evaluated whether Localized Complexity could address these issues. We hypothesized that if one used localized entropy to measure complexities, then it would capture their properties where globalized entropy failed. Thus, we next plotted Localized Spatial-Intensity Entropies and Complexities as a function of spatial scales (Equations (15) and (19)). For the sake of comparison, we present these plots for the same images as in Figure 2A–D (Figure 3). For the synthetic image, the computation was straightforward because we knew exactly what the two possible intensities were (Figure 3A,E). However, the other images had more intensities. Therefore, we had to choose the number of bins to split these intensities in the calculation of probabilities (Inequality 20). Therefore, the non-synthetic images in Figure 3 have multiple lines, each generated by a different number of bins.

The main result in Figure 3 is that the problem with Globalized Complexity observed in Figure 2 disappears with Localized Complexity at the smaller spatial scales. The problem was that Pollock’s paintings yielded less complexity than urban images. Localized Complexity yielded the opposite result at small scales as was the case for perceived complexity. This inversion was easiest to see where the blue, NBins = 6, line touched the vertical axis. This result also applied to other bins and other small scales because the lines were practically parallel. The importance of small spatial scales was that they allowed for parallel computations over more portions of the scene. However, Figure 3 also illustrates an interesting effect at large scales. For these scales, the complexity was higher for the urban image than for the artistic one. We address this issue further in the discussion (Section 5).

Another result in Figure 3 was that while the entropies fell as a power law (with coefficient of determinations $r^{2}>0.9996\pm 0.0003$; standard deviation), complexities tended to increase or be constant with spatial scales. Equations (14) and (15) suggested that without image structure, the power law would have a slope of −2 in a log-log plot. This was nearly true for the synthetic and Pollock’s images (Figure 3A,D), which yielded slopes of −1.9930±0.0008 (standard deviation) and −1.95±0.04, respectively. However, the slopes were higher than those from the urban (−1.87±0.05) and Matisse’s (−1.77±0.08) images (Figure 3B,C). The near −2 slopes for the Pollock and synthetic images were due to their statistical structures being similar across spatial scales. This similarity was apparent in the relatively flat complexity lines (Figure 3E,H). But the urban and Matisse’s images had complexities that rose with spatial scales (Figure 3F,G). Such an increase reflected images with large regions of similar properties (for example, color or intensity). This rise counterbalanced the fall of entropy, making its slope less negative.

**Figure 4:**
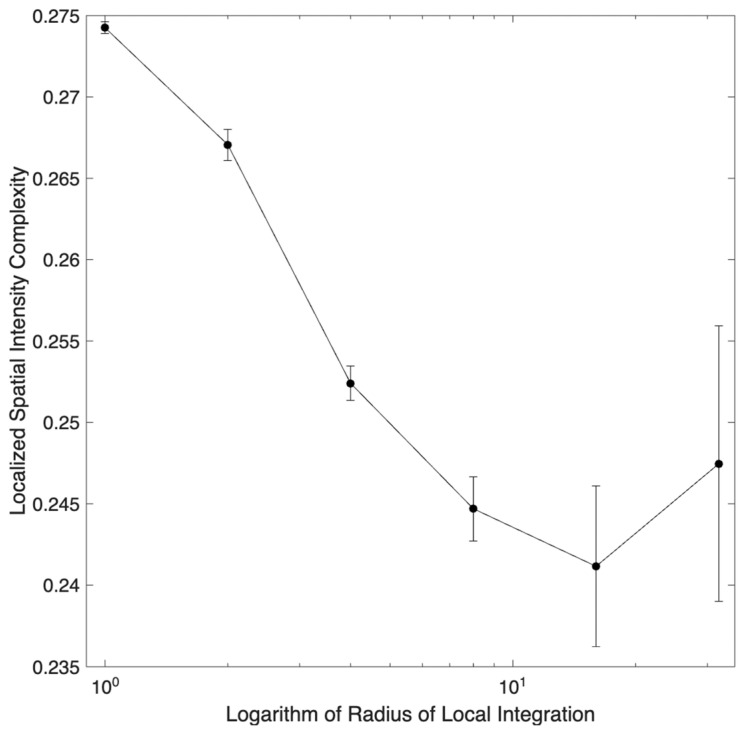
Localized spatial-intensity complexity as a function of the logarithm of the radius of local integration of synthetic images with degree of symmetry = 1. The complexity falls as a function of the radius up to 8 pixels, flattening for higher values.

We also hoped that Localized Complexity would fix the problem with the synthetic images observed with Globalized Complexity (Figure 1). Globalized Complexity could not account for the dependence of perceived complexity on the radius of local integration (Figure 1C). We thus measured Localized Complexity for synthetic images with varying radii and degree of symmetry = 1. This measurement revealed that Localized Complexity fell with increasing integration (Figure 4), thus addressing the problem with Globalized Complexity (Figure 4). The fall happened from radii of 1 to 8, flattening out for larger values.

**Figure 5:**
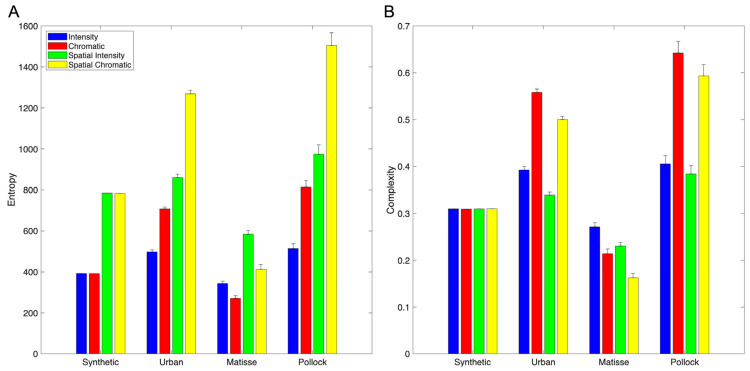
Localized entropies and complexities at spatial scale of 15. (**A**). Entropies. (**B**). Complexities. The Synthetic, Urban, Matisse, and Pollock labels correspond to Figure 2A–D, respectively. The colors of the bins are for different entropy and complexity types as indicated by the legend in Panel (**A**), and the error bars are standard errors. The results show that, as predicted, the Pollock painting has the largest entropies and complexities. In contrast, the painting by Matisse has the lowest.

Although these results showed the adequacy of Localized Spatial-Intensity Complexity at low spatial scales, the outcomes so far did not address other types of complexity. Besides Spatial-Intensity Entropies and Complexities, one could address them for first-order intensity and color distributions (Equations (14) and (18)). In addition, one could study Spatial-Chromatic Complexity instead of just intensity (Figure 1, Figure 2 and Figure 3). We thus calculated these various types of entropy and complexities at small spatial scales. Figure 5 illustrates these results for the spatial scale of 15, that is, the lowest in the bolded lines in Figure 3.

Figure 5 confirms and extends the conclusions in Figure 3 for Localized Entropies and Complexities. At the low spatial scale of 15, Pollock’s painting had the largest entropies and complexities, with the urban image coming in second. In contrast, Matisse’s painting was the least complex as expected. However, Figure 5 had other details that were of importance for modeling perceived complexity. For example, entropy and complexity were not proxies of each other. Thus, although for Pollock’s painting chromatic entropy was lower than its spatial counterparts, chromatic complexity was the largest. Another interesting example concerned the complexities of the synthetic image. All these complexities were almost identical (Figure 5B). The similarity of intensity and chromatic complexities was not surprising because these images used two intensities and two complexities combined statistically in the same manner. The spatial-complexity similarity was also not surprising for images with similar statistics across spatial scales [14]. But the spatial and non-spatial entropies were different (Figure 5A) because although the complexities were similar, the normalization constants were not (Equations (16) and (17)).

In conclusion, Localized Complexity appears to be consistent with perceived complexity at small spatial scales, with different types of localized complexity behaving differently for distinct types of images.

### 4.3. Comparison of Natural, Urban, Synthetic, and Artistic Images

**Figure 6:**
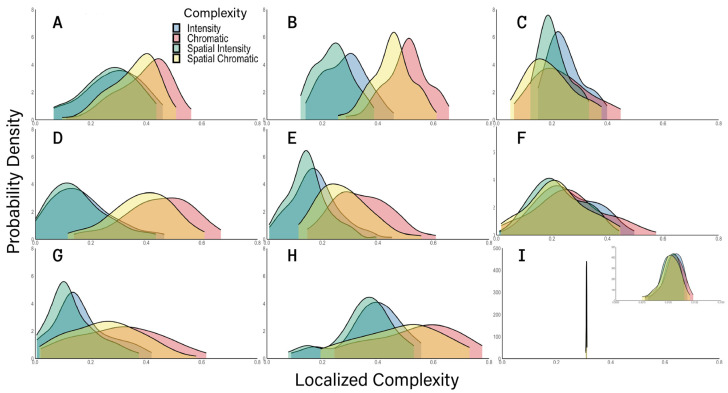
Probability densities of localized complexities across different types of images. (**A**). Natural Images from a forest near Chicago. (**B**). Urban images from small streets in Chicago. (**C**). Paintings by Giorgione. (**D**). Paintings by Claude Monet. (**E**). Paintings by Henry Matisse. (**F**). Paintings by Pablo Picasso. (**G**). Paintings by Joan Miró. (**H**). Paintings by Jackson Pollock. (**I**). Synthetic images with radius of local integration = 16 and degree of symmetry = 0.5. The transparent colors with the same conventions as in Figure 5 indicate the types of complexity (legend). Panel I includes an inset expanding the horizontal axis because localized complexities for the synthetic image have sharp distributions. The results show that chromatic variables have more complexity than luminance ones, but the details of the distributions vary by image type.

So far, we presented results for complexity in four typical examples of images (Figure 2A–D). How did these results generalize and vary across different types of images? To answer these questions, we measured Localized Complexity for natural and urban images, a variety of artistic paintings, and the synthetic images. The painters were chosen to represent different schools of art and because of their emphasis on complexity or simplicity in their work. The results of this analysis of Localized Complexity across types of images appear in Figure 6.

Figure 6 shows results that are mostly general across types of images and others that are unique. The most general result was that chromatic complexities tended to be higher than luminance ones. This difference between chromatic and luminance complexities certainly appeared to hold for natural and urban images (Figure 6A,B, respectively). We tested these differences using a Kruskal–Wallis test followed by a post hoc Mann–Whitney Test. The test rejected the null hypothesis that these differences did not exist ($p<2\times 10^{-5}$), perhaps explaining why this was also true for most artists.

However, the paintings of Giorgione, arguably the greatest landscape painter of the Renaissance, did not show the difference between chromatic and intensity complexities (Figure 6C—Kruskal–Wallis test). In contrast, Claude Monet, arguably the greatest landscape painter of Impressionism, made paintings obeying this difference (Figure 6D). We chose to study Monet because his paintings did not tend to have sharp edges [49,50,51]. We reasoned that without edges, complexities would be lower than for natural or urban images [15]. This was the case for intensity-related complexities but not for chromatic ones ($p<1\times 10^{-9}$). The same was observed for Henry Matisse’s paintings, who emphasized simplicity (Figure 6E—$p<12\times 10^{-10}$). We also expected simplicity for the cubist paintings of Pablo Picasso (Figure 6F) and Joan Miró’s late paintings (Figure 6G). For Picasso, simplicity compared to natural and urban scenes was a feature of chromatic variables ($p<4\times 10^{-11}$), and like Giorgione, the difference in chromatic and luminance complexities disappeared. The complexities of Miró’s paintings were like those of Matisse’s, with luminance producing low values compared to natural and urban pictures ($p<2\times 10^{-6}$). Finally, we worked with Jackson Pollock because his drip paintings appeared complex. This was the case for most his paintings compared with natural and urban scenes (Figure 6G—$p<2\times 10^{-4}$), except for urban colors. All these results contrasted sharply with those of the synthetic images, which produced sharp distributions of chromatic and intensity complexities (Figure 6I).

**Figure 7:**
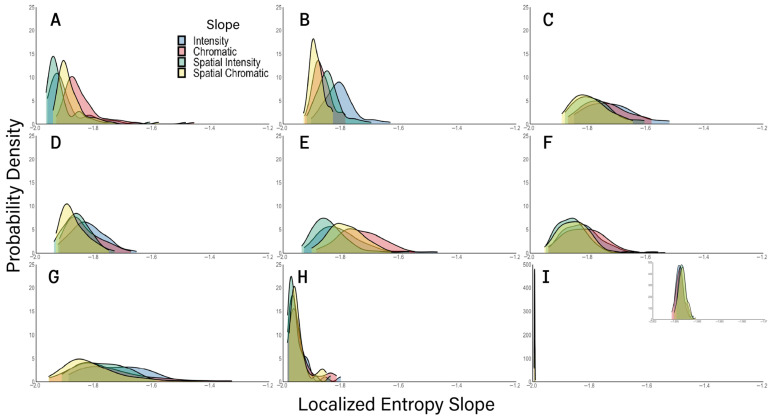
Probability densities of localized entropy slopes across different types of images. (**A**–**I**). Panels with the same conventions as in Figure 6. Slopes are less negative than −2, specially for painters emphasizing large homogenous regions like Matisse, Picasso, and Miró.

We also studied the distributions of log–log entropy slopes for the natural and urban scenes, artistic paintings, and synthetic images of Figure 6. These slopes were highly dependable, coming from straight lines with coefficient of determinations $r^{2}>0.997\pm 0.002$ (standard deviation). As described in Section 4.2, the more these slopes deviated from −2, the more their images had large regions with similar properties. Thus, these deviations of the slopes reflected image structures common to natural and urban environments and in certain artists. Figure 7 shows the distribution of these slopes in a similar format to Figure 6.

Figure 7 shows that unlike Localized Complexities (Figure 6), chromatic slopes do not tend to be systematically different from luminance slopes. For example, although natural images (Figure 7A) had less negative chromatic slopes, the opposite happened for urban images (Figure 7B). Painters were also all over the place in terms of slopes (Figure 7C–H). More interesting was how much the slopes varied from −2 for different painters. The one with the most variation was Miró, who reached the least negative slopes at more than −1.4. This reflected the large empty regions of his late paintings. Similarly, Picasso and Matisse reached slopes below −1.6, because their styles used large regions with a single color or intensity, such as in Cubism. Surprisingly, Giorgione also had such slopes. We did not expect this because as a painter from the High Renaissance, we anticipated his slopes to be “realistic,” that is, closer to those of natural and urban images [39,40]. Statistically, we ruled out the null hypothesis that the median intensity slopes of Miró and Giorgione were equal or more negative than those from natural and urban images (*p* $<7\times 10^{-8}$). Similarly, we could rule out the same hypothesis for the chromatic slopes of Matisse and Picasso (*p* <0.002). Another difference between natural and urban images and those of painters is that the distribution of slopes was sharper for the former two. Thus, painters varied the spatial structure of their art more than we observed in individual natural and urban environments.

Figure 7 also shows another important result related to Pollock’s paintings and the synthetic images. The slopes for these two types of images stayed close to −2. Statistically, we ruled out the null hypothesis that the median slopes of Pollock and the synthetic images were equal or less negative than those from natural and urban images (*p* $<7\times 10^{-8}$). In Pollock’s case, the explanation was that with dripping, he did not create images with large regions of constant properties (Figure 2D). But the explanation was different for the synthetic images because one perceived such large regions (Figure 2A). As mentioned in Section 4.1, the explanation was that embedded in the large regions were pixels with other intensities and colors. Thus, the entropy properties in small regions were like those in larger ones, being, in a sense, fractal.

Thus, complexities and spatial structures vary across different types of images, with artists not always imitating nature.

### 4.4. Is Globalized Complexity Applicable to Natural and Urban Images?

**Figure 8:**
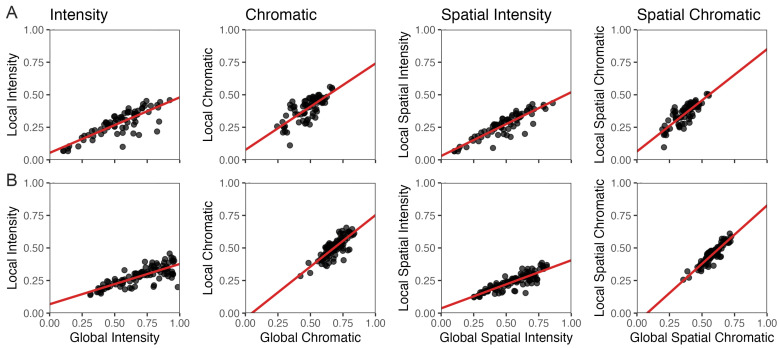
Scatter Plots for localized complexities at spatial scale 15 as a function of globalized complexities. (**A**). Natural images from a forest near Chicago. (**B**). Urban images from small streets in Chicago. The columns are for the different types of complexity (titles at the top), and the red lines are the results of linear regressions. The results indicate a linear relationship between globalized complexities and localized ones at small spatial scales. This relationship suggests that the former are good models of perceived complexity for natural and urban images.

Although globalized measures did not align with intuitive perceptions of complexity for images without large structures like Pollock’s paintings and synthetic images, perhaps such measures could do it for natural and urban scenes. To test this hypothesis, we compared Globalized and Localized Complexity for natural and urban images. Globalized and Localized complexities were computed as in Figure 1 and Figure 6, respectively. We then made scatter plots of these complexities, getting their linear regressions and coefficient of determination. These plots appear in Figure 8.

The results of Figure 8 show strong correlations between Globalized Complexities and Localized ones at low spatial scales. For a natural environment (Figure 8A), the Spearman ρ for intensity, chromatic, spatial-intensity, and spatial-chromatic complexities were 0.79, 0.75, 0.87, and 0.81, respectively. In turn, for an urban environment (Figure 8B), the same ρ were, respectively, 0.78,  0.76,  0.85, and 0.88. All these values were statistically significantly higher than 0 (*p* $<6\times 10^{-14}$). Moreover, the intercepts of the linear regressions, 0.01±0.02, were not statistically different from zero. (We did not expect the slopes to be 1 because we used the arbitrary spatial scale of 15 for Localized Complexity.)

Consequently, although Globalized Complexity does not seem consistent with perceptual complexities in general, it does for natural and urban environments.

## 5. Discussion

### 5.1. Summary

Globalized entropy, normalized or not, is not how the brain perceives complexity. Such a globalized measure has been extensively used in cognitive neuroscience because it is mathematically sensible. To get globalized complexity, one measures distributions at once for the entire image and uses each of them to get entropies. However, it is unlikely that visual processing operates through a global computation of entropy. Instead, perceived complexity seems to rely on local measurements that are subsequently integrated across space. Within this theoretical framework, localized entropy at small spatial scales appears consistent with intuitive perceptions of visual complexity across natural, urban, artistic, and synthetic images. Moreover, localized entropy seems consistent with different forms of complexity, such as those based on intensity, color, or space. Importantly, localized complexities depend on the spatial scales of the computation in a systematic manner.

Hence, our results support the two hypotheses raised in the article. The first states, “if one uses globalized entropy to measure complexities, then they would be different for some classes of images than obtained with localized entropy.” The main evidence supporting this hypothesis is that localized but not globalized entropy seems to align with perceived complexity when using the synthetic images and Pollock’s drip paintings. In turn, the second hypothesis states, “if one uses localized entropy to measure complexities, then it would capture their properties.” Our data have no aspects that contradict this hypothesis.

### 5.2. Limitations

A central limitation of the present study is the absence of direct behavioral or neurophysiological tests of our hypotheses on perceived complexity. Although the proposed localized entropy formulation is consistent with intuitive impressions of visual complexity across image classes, we have not measured well-controlled human judgment in this work. The present work should therefore be understood as theoretical and predictive in nature. Empirical tests of the predictions remain an important direction for future research.

Another limitation of our study is that so far, we have only applied our proposed measure of localized entropy to a limited set of visual variables. They have included intensity, color, and space but not, for example, time and movement. A study of movement complexity was performed recently [18], but only globalized entropy was used. Such globalized measurements might have been fine because the studied movies were from natural and urban environments. Studying the implications of our findings for time and movement may also be important for other sensory modalities. For example, sound complexity must depend on measurements of time, such as rhythm [62,63,64].

Furthermore, our study has not considered how visual balance and symmetry affect the percepts of entropy and complexity. They fall under the organizing effect of balance and symmetry [7,37,38,65,66,67,68,69,70]. However, reflection symmetry only affects localized complexity near the axis of symmetry [70]. Perhaps, the image around the axis of symmetry is all that one needs for the fall in perceived complexity if it arises from localized-complexity computations. Alternatively, symmetry may affect perceived complexity by another mechanism not studied here. For example, if a couple of samples determine that an image is symmetric, future samples could take place in just a part of the scene.

We should also mention that the definitions of complexity in this article may be too rigid. As shown elsewhere, part of the strategy of the computation of complexity is to adapt to each environment, so that we can predict how complex the next image will be [6]. Sensory adaptation is a fundamental and optimized computation of the visual system [71,72,73,74,75]. Learning is also a possibility to adapt optimally to certain types of images [76,77,78,79,80]. With learning, complexity may fall because one may have to sample images less to get the answer. Experts might only sample once or twice, whereas a first timer might sample the scene 100 times [81,82,83]. For example, if someone shows a Parisian a partial view of the Eiffel tower, they will have the image in memory and may connect the dots in their brain. But someone else might have to get many more samples. Thus, learning reduces the need for sampling because of memory. However, proposals of learning and sensory adaptation of the parameters of complexity have only been made so far based on globalized complexity [6]. Future studies will focus on how to make localized complexity compatible with learning and sensory adaptation.

Finally, we have only used 300×300 pixel images, which have lower resolution than what the visual system can do and thus, may limit our conclusions about perceived complexity. However, this low resolution does not change the main conclusion of this article. Localized Complexity at small scales appears consistent with intuitive perceptions of visual complexity. Nevertheless, the low resolution could affect Localized Complexity for some art. Artworks do not typically have the dimensions studied here. For example, Pollock and Miró oftentimes produce large rectangular artworks [84,85]. Therefore, when doing our square cropping, we tend to zoom-in on small features that may magnify the effect of small spatial scales on complexity beyond what the artist had originally desired. However, as counterargued by Allan Kaprow [84], Pollock’s artistic drip method allows for a “continuum going in all directions simultaneously, beyond the literal dimensions of any work.” Consequently, he concludes that Pollock’s technique makes the rectangular frame irrelevant.

### 5.3. Localized Versus Globalized Complexity

Why should the brain work with localized instead of globalized entropy if the latter is mathematically simpler? Part of the answer arises from neural limitations of the brain, which although has billions of neurons, each can mostly only do simple nonlinear [86,87,88] local computations [89,90,91,92,93,94]. An example is the retina, in which about a million ganglion cells [95,96,97] extract and transmit localized information to the rest of the brain. Thus, human vision never samples an image all at once. The brain uses top-down (backpropagation—[98,99]) to help put the various parts of the image together for analysis [100,101,102]. In the case of the retinal ganglion cells, their main localized mechanism is a center-surround process, extracting information on image variations (spatial, chromatic, or temporal—[103,104,105,106]). Signal variation is what entropy codes. Hence, the retina and other parts of the visual system appear to be coding a proxy of localized entropy.

More fundamentally, piecemeal (or local) computations are often easier than global ones by breaking down a complex problem into smaller, manageable subproblems [107,108,109]. This submodular approach offers five key advantages: First, it improves tractability because splitting a large system into smaller parts makes the individual computations more tractable and solvable. Second, an incremental approach can be implemented where local solutions are combined and refined in subsequent steps to approach the global optimal solution [110,111,112]. Third, the system has more memory efficiency because large, global models can require vast amounts of memory [108,113]. Fourth, the system can have parallelism, that is, individual sub-tasks running independently on different neurons or circuits (distributed computing—[114,115,116,117]). Fifth, parallelism also gives rise to redundancy such that if a local region fails, another can compensate [118,119,120,121].

But although the brain appears to compute Localized Complexity, one may use the mathematically simpler Globalized Complexity in important situations. At small scales, localized and globalized complexities exhibit strong correlations for urban and natural images. Thus, globalized complexities are applicable to these environments. Therefore, given that Globalized Complexity is easier to implement in serial machines, it may be a good proxy to predict perceived complexity in normal situations. However, we must be careful not to apply Globalized Complexity blindly to art or artificial images.

### 5.4. Complexity Versus Entropy

An important open question is which formulation is more consistent with perceived complexity: localized entropy or localized complexity? This question arises because we consider different types of entropies and complexities. The different types do not obey full rank-order correlation (Figure 5). This happens because the normalizations for spatial and non-spatial types of complexity are not the same. Therefore, in the future, one must design experiments to compare the appropriateness of entropy versus complexity. One can do these experiments with our synthetic images, by controlling different types of entropy and complexity to make one or the other larger. However, the question that one must solve first is what type of localized complexity is closest to perception. Elsewhere, we discuss how the different types of complexity may interact to give rise to a single complexity percept [6,15]. Our results suggest that the normalization makes localized complexity mathematically more fundamental than localized entropy (synthetic-image data in Figure 5). However, this does not mean that the brain implements the normalization needed for complexity.

### 5.5. The Problem of Spatial Scales

Different from globalized complexity, the localized type must include the concept of spatial scale, thus raising questions related to it. The main problem is, “if the brain uses multiple scales, how does it combine them?” The system should not emphasize a large scale as explained in Section 5.3. On the contrary, Localized Complexity seems consistent with perceived complexity at small spatial scales. We thus envision a weighed sum across small scales (within the limits of human vision), with the weights chosen genetically by evolutionary forces or chosen through learning or adaptation. The weights can also adapt to diseases and disorders of the eye and brain. For example, with sensory impairments [122,123,124,125,126], the information is noisier or distorted, possibly increasing perceived complexity at smaller scales. In another example, people with diminished visual resolution [127,128,129] may work better at larger spatial scales when measuring complexity. In the future, one should study whether perceived complexity adapts to such neural or ocular conditions. Additionally, future work could also examine how different spatial scales contribute to perceived complexity and cognitive load using behavioral and neurophysiological methods.

Another interesting issue related to spatial scales is that human perceived complexity does not differ that much once the resolution is good enough. Our work suggests that a good enough resolution for the measurement of complexity should allow for the estimation of entropy in small regions of the image. However, an open question is, “how small are these regions when the brain measures complexity?” The sizes of these regions must be determined experimentally, which is being pursued in our lab. In the meantime, because the goal of our article is to examine different theories, we have explored computationally the effects of these sizes on different images. Important results have emerged from this exploration. For example, it has revealed a surprising result in Figure 3F,H. They show that if the entropy-related spatial scale is too large, then natural and urban images may appear more complex than those by Pollock.

### 5.6. Structure in Images

Plotting localized entropy and complexity as a function of spatial scale has revealed effects of image structure. Without such structure, the localized entropy should fall as the square of spatial scale. However, log–log plots of natural, urban, and artistic images often yield slopes between −1.7 and −1.9 and as high as −1.3 instead of −2. This happens in images with largish regions with roughly constant intensity or color side by side with other areas with different but also relatively constant properties. Consequently, when the visual scales are small, subregions often fall in the largish constant areas, having low complexity. But as scales increase, the subregions begin to mix different image areas, getting more complex. Thus, the complexity is rising as a function of scale (Figure 3E–H). This rise counterbalances the slope of −2, causing the final incline to be less negative. This counterbalance is not as strong in Jackson Pollock’s art, which yields complexities that flatten at lower scales because relatively small subregions capture the overall entropy. Therefore, the relative lack of large spatial structures in Jackson Pollock’s art give rise to entropies with nearly −2 slopes.

### 5.7. Different Types of Images

Different types of images have different degrees of complexity and dependence on spatial scale. Almost all classes of images tend to have more chromatic complexity (spatial or not) than the intensity type. However, natural (forest) images are likely to have less separation between chromatic and intensities complexities than urban ones. And for Picasso and Giorgione, this separation is practically inexistent. Pollock’s paintings have the largest complexities in our study, while Matisse, Miró, and Monet have the smallest ones, specially, for luminance. These differences in complexities have two interesting implications: First, because different environments have different complexities, people may have to adapt and learn, creating distinct optimal brain models (see Section 5.2 for more discussion on this issue). Second, different artists have different aesthetic values related to complexity. Recent computational models have addressed this aesthetic individuality [13,22,130].

However, the comparison of the different types reveals one surprising complexity property that is almost universal. Localized Chromatic Complexity is higher than the Luminance type for most images. Importantly, this difference holds for everyday images, namely, the natural and urban types. The difference also holds in our study for the work of most artists, such as Monet, Matisse, Miró, and Pollock. That localized complexity shows such a difference is somewhat surprising to us because luminance is a more fundamental visual variable. The fundamental quality of luminance is seen in its contribution to a larger number of visual properties than color [19,131,132,133] and in evolutionary primacy [134,135]. The reason for luminance being more fundamental is that it is easier to measure. To get color, the visual system compares different chromatic channels, while luminance uses only one (except if one counts rods and cones to get different orders of magnitude of intensity [136,137,138]). Hence, in the tradeoff between simplicity of computation and amount of information, the former has won in evolution.

### 5.8. Does Art Imitate Nature?

Our results on complexity touch upon an issue raised by the Roman Stoic philosopher Seneca the Younger in his “*Epistulae Morales ad Lucilium*” [139]. He famously wrote, “All art is but imitation of nature.” However, if art imitates nature, then we would expect the statistics of art and nature to be similar. But their statistics are often not so. In this article, we show that complexities of art and their dependence on spatial scale are statistically significantly different from those of natural and urban images. Not only that, but these differences vary across artists. Such differences have already been pointed out in other publications [14,21,22]. These other authors have suggested that art does not imitate nature, but instead, art stimulates pleasure areas of the brain [13,140,141,142]. Sensory stimuli activate these areas when carrying good, useful information [6,13,130,143], which typically stimulates brain areas that have evolved to code it [6,13,144]. Artists can activate these areas further by exaggerating the good information. If artists do so, they cannot just imitate nature but create things that stimulate their and our own brains. If so, perhaps better than Seneca’s point is Oscar Wilde’s comeback in the *Decay of Lying* [145]. He wrote, “Life imitates Art far more than Art imitates Life … At present people see fogs, not because they are fogs, but because poets and painters have taught them the mysterious loveliness of such effects.”

## Data Availability

All data are in the Supplementary Materials reported above.

## Supplementary Materials

The following supporting information can be downloaded at: https://osf.io/nwz42 and https://sberquet.github.io/Perceived-Complexity-As-Normalized-Integrated-Local-Shannon-Entropy/, Images: Images and Data by Category: Computer Code: MATLAB Files; Statistics: Statistics Table S1, Statistics Table S2, Statistics Table S3, Statistics Table S4, Statistics Table S5, Statistics Table S6, Statistics Table S7, and Statistics Table S8.
